# Folate metabolism modifies chromosomal damage induced by 1,3-butadiene: results from a match-up study in China and in vitro experiments

**DOI:** 10.1186/s41021-021-00217-y

**Published:** 2021-10-09

**Authors:** Menglong Xiang, Zhi Wang, Peng Zou, Xi Ling, Guowei Zhang, Ziyuan Zhou, Jia Cao, Lin Ao

**Affiliations:** 1 Department of Environmental Hygiene, College of Preventive Medicine, Third Military Medical University, Chongqing, People’s Republic of China; 2 Center for Disease Control and Prevention of Northern Theater Command, Shenyang, Liaoning Province China; 3 Institute of Toxicology, College of Preventive Medicine, Third Military Medical University, Chongqing, People’s Republic of China 400038

**Keywords:** 1,3-butadiene, Folate metabolism, MTHFR, Polymorphism, Chromosomal damage

## Abstract

**Objectives:**

To explore the role of folate metabolism in 1,3-Butadiene (BD)'s genotoxicity, we conducted a match-up study in BD-exposed workers in China to analyze the associations between the polymorphisms of methylenetetrahydrofolate reductase (*MTHFR*) and the chromosomal damage induced by BD exposure, and culture-based experiments in TK-6 cells to examine the global DNA methylation levels and chromosomal damage when exposed both to BD’s genotoxic metabolite, 1,2:3,4-diepoxybutane (DEB), and MTHFR’s direct catalytic product, 5-methyltetrahydrofolate (5-MTHF).

**Methods:**

Cytokinesis block micronucleus assay (CBMN) was used to examine the chromosomal damage induced by BD or DEB. Poisson regression models were produced to quantify the relationship of chromosomal damage and genetic polymorphisms in the BD-exposed workers. Global DNA methylation levels in TK6 cells were examined using DNA Methylation Quantification Kit.

**Results:**

We found that BD-exposed workers carrying *MTHFR* C677T CC (2.00 ± 2.00‰) (FR = 0.36, 95%CI: 0.20–0.67, *P* < 0.01) or *MTHFR* C677T CT (2.87 ± 1.98‰) (FR = 0.49, 95%CI: 0.32–0.77, *P* < 0.01) genotypes had significantly lower nuclear bud (NBUD) frequencies than those carrying genotype *MTHFR* 677 TT (5.33 ± 2.60‰), respectively. The results in TK6 cells showed that there was a significant increment in frequencies of micronucleus (MN), nucleoplasmic bridge (NPB) and nuclear bud (NBUD) with exposure to DEB at each 5-MTHF dose (ANOVA, *P* < 0.01). Additionally, there was a significant decrease in frequencies of MN, NPB and NBUD in DEB-exposed cultures with increasing concentration of 5-MTHF (ANOVA, *P* < 0.05). The levels of global DNA methylation were significantly decreased by DEB treatment in a dose-dependent manner within each 5-MTHF concentration in TK-6 cells (ANOVA, *P* < 0.01), and were significantly increased by 5-MTHF supplementation within each DEB concentration (ANOVA, *P* < 0.01).

**Conclusion:**

We reported that folate metabolism could modify the association between BD exposure and chromosomal damage, and such effect may be partially mediated by DNA hypomethylation, and 5-MTHF supplementation could rescue it.

## Introduction

1,3-Butadiene (BD; CAS 106–99-0) is regarded as a potent carcinogen that is both an occupational and environmental hazard [1]. Rodent animal studies and human epidemiological studies have showed that the carcinogenicity of BD is generally attributed to the formation of DNA adducts via its reactive electrophilic metabolites: 3,4-epoxy-1-butene (EB), 3,4-epoxybutane-1,2-diol (epoxybutane diol or EBD), and 1,2:3,4-diepoxybutane (DEB). Among them, DEB exerts the utmost genotoxic potency, roughly 100 times stronger than EB [2]. Our group have performed a series of studies in a population with occupational exposure to BD and found that BD exposure was associated with the increased genotoxic damage for multiple endpoints in peripheral blood lymphocytes of workers, including HPRT mutation and formation of micronucleus (MN), nucleoplasmic bridge (NPB) and nuclear bud (NBUD) [3, 4].

Recent studies have indicated that BD is also an epigenotoxic agent. Short-term exposure to BD leads to a variety of epigenetic alterations in the liver of mice, such as global DNA hypomethylation [5–7]. Meanwhile, a growing body of evidence supports the hypothesis that epigenetic alterations may serve as early indicators of exposure to mutagens or carcinogens and function as an interface between the inherited genome and the environment [8, 9]. For instance, alterations in DNA methylation may lead to reactivation of oncogenes and repetitive DNA sequences, to silencing of tumor-suppressor genes, and can result in genomic instability and cancer [10]. The relationship of DNA methylation and DNA damage in the mutagenesis of BD is worthy of further study.

Folate metabolism, for its critical role in maintaining genomic stability by regulating DNA biosynthesis, repair and methylation, could be a research focus on linkage of DNA methylation and genetic damage induced by mutagens. It is well established that folate deficiency can lead to chromosomal damage, such as micronucleus formation, due to the deficient methylation of dUMP to dTMP, and subsequently, incorporation of urail into human DNA [11]. On the other hand, there are some observations suggesting that altered folate status also modulates the epigenome, i.e., DNA methylation [12, 13]. Therefore, we reasoned that the association of DNA damage and non-genotoxic epigenetic alterations in response to BD exposure may be accounted for by folate metabolism.

In the process of folate metabolism, methylenetetrahydrofolate reductase (MTHFR) is a key enzyme which catalyses the irreversible conversion of 5,10-methylenetetrahydrofolate (5,10-MTHF, the methyl donor in the conversion of dUMP to dTMP) into 5-MTHF. Then, 5-MTHF remethylates homocysteine to methionine, which is further metabolized to S-adenosylmethionine (SAM) as the most universal methy donor in cells. This key protein, therefore, controls whether folate is partitioned towards DNA precursor synthesis or DNA methylation [13]. Two most frequent mutations of *MTHFR* are the single-nucleotide polymorphisms (SNPs), C677T and A1298C. The two enzymatic isoforms have a weaker capacity, which can reach − 75%, to generate 5-MTHF, but C677T can cause worse handicaps [14]. Human epidemiological studies showed that the common 677C > T polymorphism in the gene encoding MTHFR could modify associations between folate status and genomic stability in cells [15]. Besides, Kakkoura, M. G., et al. found that serum levels of 5-MTHF were shown to be influenced by interactions between *MTHFR* (rs1801133) polymorphisms and Mediterranean diet [16]. Interestingly, an earlier study also reported *MTHFR* polymorphisms were associated with DNA hypomethylation [17]. Therefore, we reasoned that folate metabolism mediated by MTHFR might be involved in the linkage of genomic instability and epigenotoxic response induced by BD.

The present study aimed to elucidate the critical role of folate metabolism in the mechanism of BD-induced toxicity in cells: both in genotoxic and epigenotoxic level. We examined the impact of *MTHFR* polymorphisms on chromosomal damage in a population of BD-exposed workers and adopted cell culture-based experiments with human lymphoblastoid cells (TK6) cells to explore the relationship of DNA methylation and genetic damage induced by DEB, a genotoxic metabolite of BD in human body.

## Materials and methods

### Study population

As described earlier, we conducted a 1:1 matched pair study at a petrochemical product company in the Nanjing area of China [3]. Forty-five BD-exposed workers paired with an appropriate control from the same plant were recruited for the study and matched by gender, smoking habits, and close age (±3 years). Questionnaires for all the subjects were accompanied by regular physical examinations at the Yangzi Employee Hospital. Meanwhile, blood samples were collected for further study. An informed consent was obtained from each subject at the start of this study.

### Chemicals

DEB was purchased from Sigma Aldrich Company, U.S., and Cytochalasin-B (Cyt-B) was purchased from Solarbio Company, China, and 5-methyltetrahydrofolate (5-MTHF) was purchased from TRC-Canada. DEB, Cytochalasin-B and 5-MTHF solutions were made in dimethyl sulfoxide (DMSO, Sigma Co., U.S.), and stored at − 80 °C.

### Cell culture

The human B lymphoblastic cell lines (TK-6 cells) were prepared at a concentration of 2 × 10^5^ cells/ml in 5 ml of RPMI 1640 folic acid-free medium (Lot:1694540, Gibco) containing 3 nmol/L, 30 nmol/L or 300 nmol/L 5-MTHF, respectively, and 10% fetal bovine serum (Lot: 42F3352K, Gibco). 5-MTHF, which is the direct catalytic product of the MTHFR enzyme, ranged from the deficiency to sufficiency concentration (3 ~ 300 nmol/L) to partially simulate different MTHFR activities, with 3 nmol/L as the lower activity, 30 nmol/L as the normal activity, and 300 nmol/L as the intervention group, respectively. The cultures were incubated at 37 °C and 5% CO_2_ for 24 h, then each 5-MTHF group was exposed to DEB at 4 dose levels (at a final concentration of 0 μmol/L, 10 μmol/L, 20 μmol/L or 40 μmol/L, respectively, and for the 0 μmol/L group, cells were treated with DMSO solely as control) in triplicates.

### CBMN assay

We have described the CBMN assay conducted in the lymphocytes of BD-exposed workers in earlier studies [3]. For TK-6 cells, after 24 h exposure of 5-MTHF and DEB, the cell cultures were refreshed by new culture medium, then added with Cyt-B at a final concentration of 4.5 μg/ml, incubated for another 28 h. The cells were harvested by centrifugation and fixed in methanol: acetic acid (3:1) after hypotonic treatment with 0.075 M KCl. Then, a cell suspension was directly dropped onto clean slides. Slides were air-dried and stained with Giemsa (Sigma–Aldrich). In both experiments, one thousand bi-nucleated lymphocytes per subject were scored blindly by a single investigator for the presence of MNi, NPBs and NBUDs. The MNi, NPBs and NBUDs frequencies were the number of MNi, NPBs and NBUDs observed per 1000 lymphocytes, expressed as a count per thousand (‰).

### *MTHFR* genotyping

Genomic DNA of the BD-exposed workers was directly extracted from EDTA-anticoagulated whole blood using a wizard genomic DNA purification kit (Promega Corp., Madison, WI, USA) according to the manufacturer’s instructions. PCR-restriction fragment length polymorphism (PCR-RFLP) was the main genotyping method employed. PCR-RFLP for *MTHFRC677T* and *A1298C* were performed under the following conditions: 94 °C for 10 min was followed by 38–40 cycles of 94  for 1 min, 60.5 °C for 1 min and 72 °C for 1 min and elongation at 72 °C for 10 min. PCR products were digested with specific restriction enzymes that recognized and cut either at the wild-type or variant sequence site. Primers and restricted endonucleases were shown in Table 1.

**Table 1:** PCR primers and restricted endonucleases for *MTHFR* gene in genotyping process

SNP	Primers		PCR method	Restricted endonucleases
	Forward	Reverse		
*MTHFR 677*	5′-TGAAGGAGAAGGTGTCTGCGGGA-3′	5′-AGGACGGTGCGGTGAGAGTG-3’	RFLP	Hinf1
*MTHFR 1298*	5′-CTTTGGGAGCTGAAGGACTAC-3’	5′-CACTTTGTGACCATTCCGGTTTG-3’	RFLP	Mbo2

### Analysis of global DNA methylation

The total genomic DNA was extracted from the TK-6 cells using the DNA isolation Kit (Promega, Madison, WI, USA) according to the manufacturer’s instructions. The genome-wide methylation level was detected by DNA Methylation Quantification Kit (Epigentek, New York, NY, USA) following the manufacturer’s protocol. The analysis provides the levels of global DNA methylation, and it is not specific to any particular gene. The data were presented in terms of percent of control (DMSO-exposed TK-6 cells). Experiments were carried out in triplicate.

### Statistical analysis

For the epidemiological study concerning BD-exposed workers, Poisson regression models as described by Wang, et al [18] were produced to quantify the relationship of chromosomal damage and the genotypes or diplotypes, estimated by the frequency ratio (FR) (FR = e^β^, e = 2.71828 β:regression coefficient) with 95% confidence intervals. FR was adjusted for age, sex, smoking status, alcohol drinking in a multivariate Poisson regression analysis. For categorical variables, the FR indicated a proportional increase/decrease of the MN/NPB/NBUD frequency in a comparison group relative to the reference. For TK-6 cells experiments, all results were expressed as the mean ± SE. One-way ANOVA repeated measures test was used to determine the effect of 5-MTHF dose on the parameters measured in control and DEB-treated cultures. Associations between MN, NPB and NBUD frequency were studied using Pearson correlation analysis. Two-way ANOVA was used to determine both the independent effect of 5-MTHF and DEB as well as to test whether there was a significant interaction between these variables. Statistical analyses were performed using SAS 9.0(SAS Institute Inc., USA) and SPSS 17.0 (SPSS Inc., USA).

## Results

### Chromosomal damage and distribution of genotypes in BD-exposed workers

As described before, the match-up process resulted in 45 pairs of subjects. We found that the pairs were well matched for baseline information, such as gender, age and smoking habits, with a mean age of 40.6 in both of the BD-exposed group and the control group. The data on the chromosomal damage in BD-exposed workers and controls shows that, briefly, the numbers of MNi and NPBs frequency were significantly higher (*P* < 0.01) in BD-exposed workers than in the control subjects, respectively [3]. The allele frequencies of each single nucleotide polymorphism site of *MTHFR* were showed in Table 2. The genotype distributions at each locus were consistent with the Hardy–Weinberg equilibrium.

**Table 2:** Distribution of *MTHFR* genotypes and allele frequencies among 1, 3-butadiene (BD) exposed workers

SNPs	Genotypes	N	Rate%	Frequency
*MTHFR*677	CC	13	28.9	C:0.53
	CT	22	48.9	T:0.47
	TT	10	22.2	
*MTHFR*1298	CC	2	4.4	C:0.22
	CA	16	35.6	A:0.78
	AA	27	60.0	

#### Polymorphism analysis of MTHFR gene

In multivariate Poisson regression model, we found that BD exposed workers carrying *MTHFR* C677T CC (2.00 ± 2.00‰)(FR = 0.36, 95%CI: 0.20–0.67, *P* < 0.01) or *MTHFR* C677T CT (2.87 ± 1.98‰)(FR = 0.49, 95%CI: 0.32–0.77, *P* < 0.01) genotypes had significantly lower NBUD frequencies than those carrying genotype *MTHFR* 677 TT (5.33 ± 2.60‰), respectively (Tables 3 and 4). However, none of genotypes of *MTHFR* A1298C were identified to impact chromosomal damage in BD-exposed workers.

**Table 3:** Chromosomal damage between genotypes of *MTHFR* in BD-exposed workers

Gene	N	MN(‰)	NPB(‰)	NBUD(‰)	NDI
*MTHFR* C677T					
CC	11	7.73 ± 4.38	2.73 ± 3.61	2.00 ± 2.00*	2.22 ± 0.11
CT	23	7.61 ± 3.29	2.96 ± 2.84	2.87 ± 1.98*	2.22 ± 0.12
TT	9	8.89 ± 4.62	1.56 ± 1.33	5.33 ± 2.60	2.13 ± 0.12
*MTHFR* A1298C					
AA	28	8.21 ± 3.91	2.07 ± 2.80	3.21 ± 2.50	2.19 ± 0.14
AC	14	7.50 ± 3.78	3.64 ± 2.76	3.07 ± 2.20	2.22 ± 0.11
CC	1	5	3	3	2.13
AC/CC	15	7.33 ± 3.70	3.60 ± 2.67	3.07 ± 2.12	2.21 ± 0.14

*NDI* nuclear division index

*As seen in Table 4, compared in the BD-exposed group, *P* < 0.01

**Table 4:** Multivariate poisson regression analysis for association between genetic polymorphism (*MTHFR*) and chromosomal damage in BD-exposed workers

Name	Β	95%CI		X^2^	P	FR
		low	Upper			
Intercept	1.6065	0.8771	2.3360	18.63	<.0001	
Gender (female)	0.3131	−0.2650	0.8911	1.13	0.2884	1.37 (0.77–2.44)
Age(≤40)	0.1725	−0.3341	0.6790	0.45	0.5046	1.19 (0.72–1.97)
OL(≤20)	−0.2213	−0.7090	0.2663	0.79	0.3736	0.80 (0.49–1.31)
Smoking (no)	−0.1197	− 0.6219	0.3826	0.22	0.6405	0.89 (0.54–1.47)
Drinking (no)	−0.2699	− 0.7513	0.2116	1.21	0.2720	0.76 (0.47–1.24)
*MTHFR*677(CC)	−1.0185	−1.6335	−0.4036	10.54	0.0012	0.36 (0.20–0.67)*
*MTHFR*677(CT)	−0.7055	−1.1513	−0.2597	9.62	0.0019	0.49 (0.32–0.77)*
*MTHFR*1298(AC/CC)	0.2778	−0.1801	0.7357	1.41	0.2344	1.32 (0.84–2.09)

*Compared in BD-exposed group, *MTHFR* 677 TT genotype as reference, *P* < 0.05. OL: Occupational Longevity

### Effects of DEB exposure and 5-MTHF addition on chromosomal damage in TK-6 cells

We adopted DEB, one of the metabolites of BD, to treat TK6 cells, and found that there was a significant increment in frequencies of MN, NPB and NBUD with exposure to DEB at each 5-MTHF dose (ANOVA, *P* < 0.01). Additionally, there was a significant decrease in frequencies of MN in all of DEB-exposed cultures with increasing concentration of 5-MTHF (ANOVA, *P* < 0.05) (Table 5). The frequencies of NPB were significantly decreased in 20 μmol/L (ANOVA, *P* = 0.014) and 40 μmol/L (ANOVA, *P* < 0.001) DEB-exposed cultures and the frequencies of NBUD decreased significantly only in 40 μmol/L (ANOVA, *P* < 0.001) DEB-exposed cultures, depending on 5-MTHF concentrations, respectively. The decrease in frequencies of NPB and NBUD in the 300 nmol/L 5-MTHF cultures relative to the 3 nmol/L 5-MTHF cultures was significant for the 10 μmol/L DEB-exposed cultures (t-test, *P* < 0.05), respectively. Two-way ANOVA analysis showed that there was a significant interaction between 5-MTHF and DEB with respect to observed MN, NPB and NBUD frequency (*P* interaction< 0.001). A significant positive correlation between MN, NPB and NBUD frequency was observed in the DEB-exposed cultures (Pearson correlation, MN and NPB, *P* < 0.001, *R*^*2*^ = 0.953; MN and NBUD, *P* < 0.001, *R*^*2*^ = 0.938; NPB and NBUD, *P* < 0.001, *R*^*2*^ = 0.957. *n* = 36).

**Table 5:** Effects of DEB exposure and 5-MTHF addition on chromosomal damage in TK-6 cells

		5-MTHF (nmol/L)			One-way ANOVA *P*	Two-way ANOVA *P* interaction
CBMN markers	DEB (μmol/L)	3	30	300		
MN(‰)	0	7.00 ± 1.00	7.33 ± 1.528	4.00 ± 2.646	0.129	
	10	21.67 ± 1.16^aa^	13.67 ± 2.31^**^	12.33 ± 1.16^***aa^	0.001	
	20	31.00 ± 1.00^aaabb^	25.00 ± 1.00^*aaabb^	20.67 ± 4.51^**aaabb^	0.010	
	40	59.33 ± 3.79^aabc^	35.67 ± 7.02^**aaabbbcc^	29.33 ± 1.53^***aaabbbcc^	< 0.001	
	One-way ANOVA *P*	*<* 0.001	*<* 0.001	*<* 0.001		*<* 0.001
NPB(‰)	0	4.00 ± 1.00	3.00 ± 1.00	1.33 ± 0.58^*^	0.027	
	10	6.00 ± 1.00	5.33 ± 2.08	2.66 ± 1.53^*^	0.092	
	20	12.67 ± 1.53^aaabb^	9.33 ± 1.53^*aab^	7.33 ± 1.53^**aabb^	0.014	
	40	25.00 ± 2.65^aaabbbccc^	17.33 ± 1.53^**aaabbbccc^	12.00 ± 1.73^***#aaabbbcc^	< 0.001	
	One-way ANOVA *P*	*<* 0.001	*<* 0.001	*<* 0.001		*<* 0.001
NBUD(‰)	0	8.00 ± 1.00	6.67 ± 1.16	3.67 ± 0.58^**##^	0.004	
	10	11.00 ± 1.00^a^	10.67 ± 2.08	7.33 ± 1.53^*#aa^	0.056	
	20	13.33 ± 1.53^aa^	12.00 ± 2.646^a^	12.00 ± 1.00^aaabb^	0.621	
	40	27.33 ± 1.53^aaabbbccc^	20.67 ± 2.52^**aaabbbcc^	15.67 ± 1.16^***#aaabbbcc^	< 0.001	
	One-way ANOVA *P*	*<* 0.001	*<* 0.001	*<* 0.001		*<* 0.001

ANOVA, compared with 5-MTHF 3 nmol/L,*p < 0.05, **p < 0.01, ***p < 0.001; compared with 5-MTHF 30 nmol/L,#*p <* 0.05, ##*p <* 0.01, ###*p <* 0.001. Compared with DEB control (0 μmol/L), a, *p <* 0.05, aa, *p <* 0.01, aaa, *p <* 0.001; compared with DEB 10 μmol/L, b, *p <* 0.05, bb, *p <* 0.01, bbb, *p <* 0.001; compared with DEB 20 μmol/L, c, *p <* 0.05, cc, *p <* 0.01, ccc, *p <* 0.001

### Effects of DEB exposure and 5-MTHF addition on the extent of global DNA methylation in TK-6 cells

**Fig. 1:**
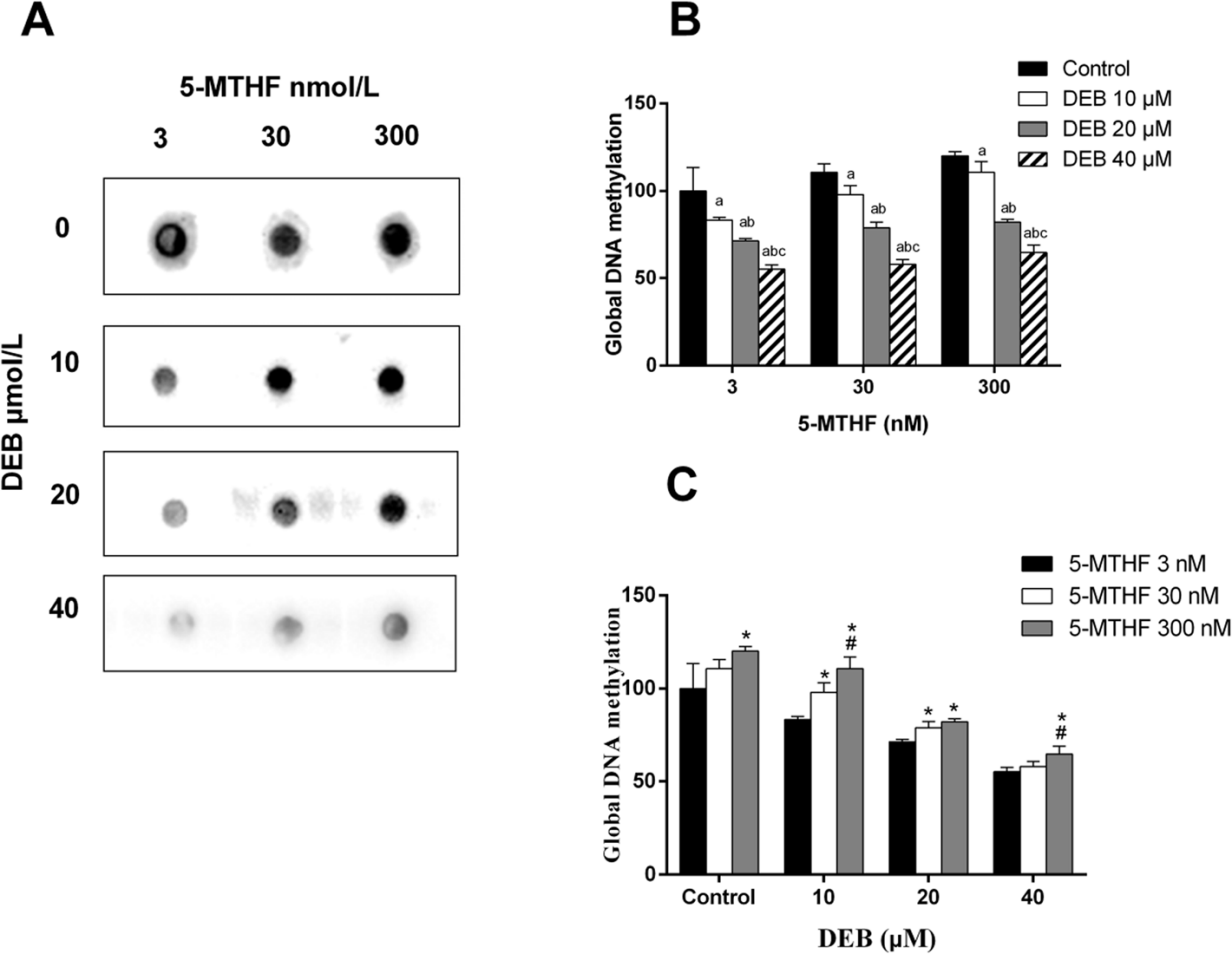
Effects of DEB exposure and 5-MTHF addition on the extent of global DNA methylation in TK-6 cells. **A** Typical images of global DNA methylation test in TK-6 cells; **B** The level of global DNA methylation within each 5-MTHF concentration; **C** The level of global DNA methylation within each DEB concentration; (Within each 5-MTHF concentration, a, compared with DEB control group, *p* < 0.05; b, compared with DEB 10 μmol/L group, *p* < 0.05; c, compared with DEB 20 μmol/L group, *p* < 0.05. Within each DEB concentration, *, compared with 5-MTHF 3 nmol/L group, *p* < 0.05; #, compared with 5-MTHF 30 nmol/L group, *p* < 0.05)

The epigenetic responses to DEB exposure and 5-MTHF addition in TK-6 cells were also evaluated, as reflected by global DNA methylation level (Fig. 1A). Figure 1 showed that levels of global DNA methylation were significantly decreased by DEB treatment in a dose-dependent manner within each 5-MTHF concentration in TK-6 cells, and were significantly increased by 5-MTHF supplement within each DEB concentration (Fig. 1B, C). Two-way ANOVA analysis showed that there was a significant interaction between 5-MTHF and DEB treatment with respect to observed global DNA methylation level (*P* interaction =0.003).

## Discussion

The present study showed that the polymorphisms of *MTHFR* may have a significant impact on chromosomal damage in BD-exposed workers. There exist two common low-function polymorphic variants of *MTHFR*: the T variant at nucleotide 677 (*MTHFR* C677T) and the C variant at nucleotide 1298 (*MTHFR* A1298C). The first of these variants, C677T, has been shown to be associated with higher baseline homocysteine levels in the serum and is associated with increased risk of vascular disease and neural tube defects, and polymorphisms of A1298C may be risky factors of multiple tumors in several studies. In 2008, a study in Beijing area [19] found that BD-exposed workers carrying *MTHFR* 1298 AA genotypes had significantly lower MN frequencies (6.16 ± 5.06)‰ than those carrying AC or AC/CC genotypes[(8.12 ± 5.58)‰, *P* = 0.03]. In further haplotype analysis of *MTHFR* gene, the individuals in BD-exposed group carrying diplotype 677C-1298A/ 677C-1298A had significantly lower MN frequencies than those carrying diplotype 677 T -1298A/ 677 T-1298C, which indicates that *MTHFR* C677T T variant contributes mainly to the interactions between *MTHFR* haplotypes and chromosomal damage. The present study included both of SNP loci in *MTHFR* gene, and the result showed that BD-exposed workers carrying *MTHFR* C677T C allele had significantly lower NBUD frequencies than those carrying TT genotype, which validated that *MTHFR* C677T T allele may play a pronounced role in human genetic susceptibility towards BD-induced mutagenicity. Most of *MTHFR* polymorphism studies focus on associations with cancer or metabolic diseases. How interactions between environmental chemicals and *MTHFR* genetic polymorphisms could influence genetic damage attracts interest in a few of studies. For example, several studies concerning air pollution among traffic policemen or railroad transit workers, in which airborne BD is a major pollution component [20, 21], suggested that *MTHFR* gene polymorphisms were associated with individual chromosomal damage. Our results, consistent with these studies, raises the concerns that the effect of occupational exposure to BD may be significantly modulated by folate status which varies greatly amongst individuals. An in vitro study suggested that heterozygous (CT) or homozygous (TT) genotype for this polymorphism represents a reduced enzyme activity of 65 and 30% of wild-type (CC), respectively [8]. Individuals carrying *MTHFR* C677T TT genotype have been reported to have lower levels of 5-MTHF, probably resulting in lower cell methionine and S-adenosylmethionine (SAM) levels, thereby potentially leading to aberrant DNA methylation [22, 23], which, we hypothesized, may explain the increased NBUD frequencies in BD-exposed workers in the present study.

Therefore, we further conducted cell-cultured experiments to investigate the role of folate metabolism in BD-induced toxicity. In all three genotoxic metabolites of BD, the most consistently positive genotoxic effects have resulted from treatments with DEB in a number of studies. One study reported that DEB is 25- and 50-fold more effective than EB and EBD, respectively, when inducing SCEs in the PBLs of healthy individuals [2]. Thus we chose DEB to simulate BD-induced toxicity in human body. Under the treatment of DEB with a range of concentrations (10 ~ 40 μmol/L), the chromosomal damage and the global DNA methylation levels of TK-6 cells were detected. The results showed that, within each 5-MTHF concentration, the frequencies of multiple genotoxic endpoints (MNi, NPBs and NBUDs) significantly increased and the global DNA methylation levels significantly decreased in a dose-related manner with DEB exposure, which indicated that DEB exposure were highly associated with both of status in TK-6 cells. Evidence supporting epigenetic dysregulation as a model of exogenous genotoxic compounds is mounting. A number of mutagenic chemicals, such as 2-acetylaminofluorene, tamoxifen, trichloroethylene, aflatoxin B1, ochratoxin, nickel, chromiumare known to form adducts and to induce epigenetic distortions in DNA [24]. A study examined a panel of genetically diverse inbred mice under the BD exposure and observed loss of global DNA methylation as well as DNA damage in C57BL/6 J mice [6]. Our study in TK-6 cells is in agreement with these studies. However, whether DNA hypomethylation occurs as a result of DEB-induced DNA damage or vice versa remains unclear. There is possibility that epigenetic regulation and DNA damage/repair process act reciprocally. Recent advances in the field of research on epigenetic mechanisms of carcinogenesis indicate that epigenetic changes may occur either as a consequence of DNA damage or a non-genotoxic mechanism for it [25].

Folate deficiency has been associated both with increased DNA damage and aberrant DNA methylation in vitro and in vivo [26]. Disruption of the normal methyl group metabolism (5-MTHF, methionine, and subsequent SAM) caused by folate depletion can directly modify DNA epigenetic patterns. In our study, when the TK-6 cells were only treated with 5-MTHF (the DEB sham exposure groups), the global DNA hypomethylation state was significantly rescued by the 5-MTHF addition, which confirmed that 5-MTHF could negate the DNA hypomethylation enhanced by folate depletion. There is good evidence suggesting that folate depletion can generate low cellular 5,10-MTHF, then bring about elevated uracil misincorporation into DNA and decreased de novo biosynthesis of purines and thymidylate, finally result in severe DNA damage [12]. It is worthy to note that the protective effect of 5-MTHF on chromosomal damage in these 5-MTHF-only groups was less pronounced, only the frequencies of NPB and NBUD were significantly reduced in the highest concentration of 5-MTHF (300 nmol/L). Interestingly, within each DEB concentration, both the chromosomal damage and the global DNA hypomethylation of TK-6 cells exhibited a dose-response response to 5-MTHF supplementation. Besides, further analysis indicated a significant interaction between 5-MTHF and exposure to DEB. Taken together, our data suggested that the protective effect of 5-MTHF supplementation toward chromosomal damage became more obvious only under the exposure of DEB. For the indirect role of 5-MTHF in DNA damage and repair process, we assumed that the most possible mechanism of such protective effect was through regulation of DNA methylation. This, in turn, suggested that the chromosomal damage induced by DEB exposure was, at least, partially due to the DNA hypomethylation caused by DEB. Because if DNA hypomethylation was a consequence of DNA damage induced by DEB, negation of global DNA hypomethylation by 5-MTHF would not rescue the DNA damage level. However, the exact mechanism warrants further investigation.

Convincing evidence exists has also highlighted the role of oxidative stress in the linkages between folate metabolism and DNA methylation. Oxidative stress inducers have been found to decrease folate levels in cells. A study reported that an oxidative stress decreased folate synthesis by the bacteria, which would lead to folate deficiency for the host [27]. On the other hand, folate deficiency or disorders of folate metabolism pathways can induce oxidative stress, and folate supplementation can decrease the level of oxidative stress, and rescue the hypomethylation state of genome. A study found that folate and vitamin B12 supplementation decreased the level of oxidative stress and ameliorated the cytotoxic effects of AOM [28]. A recent study also found that folate and B12 treatment attenuated oxidative stress, decreased DNA total methylation levels and increased methylation level of SORBS1 induced by homocysteine [29]. Thus, it is evident that oxidative stress is closely associated with disorders of folate metabolism and methyl donor production pathways. Considering that it has been clarified that BD and its metabolites can cause oxidative stress damage in cells, whether BD and its metabolites-induced oxidative stress are involved in mediating its methylation-regulated effects deserves in-depth investigation.

It is worth mentioning that in cell experiments, all three endpoints of CBMN (MN, NPB and NBUD) were induced by DEB exposure, while in the BD-exposed workers, only the effect in the NBUD frequency was observed when evaluating the association between *MTHFR* polymorphism and chromosomal damage. In our previous studies, we found that MNi and NPBs frequencies differed significantly between BD-exposed workers and the controls, but no such response was observed for NBUDs frequencies. The polymorphisms of DNA repair genes (*XRCC1*) were found to be associated with NBUD frequencies in the BD-exposed workers [30]. The linkage between NBUD frequency and *MTHFR* polymorphisms found in the present study were similar with that. NBUD is regarded as a biomarker for the DNA repair process, which involves both the DNA repair genes (XRCC1) and the folate metabolic genes (MTHFR). Our results, taken together, provide some evidence that NBUD frequencies should be monitored in occupational exposure studies, especially when considering genetic susceptibility in workers. The differences of NBUD frequency changes between human subjects and cell samples may also reflect the differences between epidemiological studies and cell experiments. The human epidemiological study in the present study has some inherent limitations: some confound factors could not be excluded; low exposure levels may cause weak effects hard to investigate; small sample sizes, and so on. The cell experiments have some advantages above that, for example, higher exposure levels, so it is understandable that the TK6 cells exposed by DEB exhibited increased frequencies of all of MN, NPB and NBUD.

## Conclusion

In conclusion, we reported that folate metabolism could modify the association between BD exposure and chromosomal damage, as reflected by *MTHFR* polymorphism analysis in BD-exposed workers. The results of experiment conducted in TK-6 cells support the hypothesis that the chromosomal damage in response to BD exposure may be mediated by DNA hypomethylation, and 5-MTHF supplementation could rescue such effect. However, further research is needed to clarify the detailed mechanism of the interaction between DNA damage and genomic epigenetic patterns in BD’s toxicity. Our finding also proposed that folate supplementation for the BD-exposed workers may help to reduce the long-term hazardous effects from this toxic substance, which needs to be further validated in future studies.

## Data Availability

All generated data are included in this manuscript.

## Abbreviations

BD: 1,3-Butadiene
DEB: 1,2:3,4-diepoxybutane
MTHFR: Methylenetetrahydrofolate reductase
CBMN: Cytokinesis block micronucleus assay
MN: Micronucleus
NPB: Nucleoplasmic bridge
NBUD: Nuclear bud
5-MTHF: 5-methyltetrahydrofolate
5,10-MTHF: 5,10-methylenetetrahydrofolate
EB: 3,4-epoxy-1-butene
EBD: 3,4-epoxybutane-1,2-diol
FR: Frequency ratio
RFLP: Restriction fragment length polymorphism
NDI: Nuclear division index
